# Roles of miR‐181 Family Members in OSCC: Implications for Therapy and Diagnostics

**DOI:** 10.1002/cam4.71266

**Published:** 2025-09-26

**Authors:** Alexandra Iulia Aghiorghiesei, Andreea Nutu, Nikolay Mehterov, Christos K. Kontos, Boyan Vladimirov, Rares Buduru, Cornelia Braicu, Ioana Berindan‐Neagoe

**Affiliations:** ^1^ Department of Prosthodontics and Dental Materials Faculty of Dental Medicine, Iuliu Hațieganu University of Medicine and Pharmacy Cluj‐Napoca Romania; ^2^ Research Center for Functional Genomics, Biomedicine and Translational Medicine Iuliu Hațieganu University of Medicine and Pharmacy Cluj‐Napoca Romania; ^3^ Department of Medical Biology Medical University‐Plovdiv Plovdiv Bulgaria; ^4^ Research Institute Medical University‐Plovdiv Plovdiv Bulgaria; ^5^ Department of Biochemistry and Molecular Biology, Faculty of Biology National and Kapodistrian University of Athens Athens Greece; ^6^ Department of Maxillofacial Surgery Medical University‐Plovdiv Plovdiv Bulgaria; ^7^ Clinical Unit of Maxillofacial Surgery University Hospital Pulmed Plovdiv Bulgaria; ^8^ Stomestet Clinic Cluj‐Napoca Romania; ^9^ Doctoral School The Iuliu Hatieganu University of Medicine and Pharmacy ‐Cluj‐Napoca Cluj‐Napoca Romania; ^10^ Medical Sciences Academy Bucharest Romania

## Abstract

Oral squamous cell carcinoma (OSCC) is a common and aggressive form of head and neck cancer, frequently associated with smoking, alcohol consumption, and HPV infection. MicroRNAs (miRNAs) are small, non‐coding RNA transcripts that play significant roles in cancer initiation and progression. In this study, we focused on the miR‐181 family members' influence on OSCC tumorigenesis and progression, focusing on their distinct biological functions and regulatory mechanisms in OSCC‐specific contexts. Attention was given to the potential of these transcripts as biomarkers, considering their differential expression in OSCC tissues and biofluids such as plasma, serum, and saliva. Alterations in salivary miR‐181 levels have been correlated with different stages of oral lesion progression, underscoring their utility as non‐invasive biomarkers for early detection and risk stratification. Moreover, we discuss the implications of miR‐181 family modulation on biological processes in preclinical OSCC studies, highlighting their involvement in cancer hallmarks, including invasion, migration, metastasis, radio‐ and chemotherapy resistance. These findings underscore the therapeutic and diagnostic potential of the miR‐181 family in OSCC management.

## 1 Introduction

Oral squamous cell carcinoma (OSCC) represents around 90% of all types of oral cancer and, when combined with pharyngeal cancer, ranks as the sixth most common cancer globally [1]. For developing countries, the incidence is much higher, making it the third most common cancer [2]. OSCC most frequently involves the tongue, followed by the floor of the mouth, the gingiva, and the alveolar mucosa. A high incidence of tumor metastasis (40%–60%) and recurrence is observed after treatment, while around 30%–50% of patients survive longer than 3 years [3]. The relatively low survival rate is often attributed to late diagnosis [4]. Therefore, there is an urgent need to discover new biomarkers and treatment options for OSCC based on a better comprehension of the molecular mechanisms of its formation and development, in which an important role is played by the regulation of coding and non‐coding gene expression [1].

miRNAs are endogenous, small (20–22 nucleotides in length) noncoding RNA molecules. These short‐length transcripts proved to have an essential role in regulating gene expression. They directly impact essential signaling and network modulation, leading to wide‐ranging pathophysiological and pathological mechanisms that affect cell fate [5]. miRNAs primarily interact with the 3′ untranslated regions (3′ UTRs) of target mRNAs to suppress gene expression. However, studies have also reported miRNA binding to other regions, including the 5′ UTR, coding sequences, and even gene promoters, suggesting a broader regulatory role beyond post‐transcriptional repression [6]. The “seed region,” namely the first two to eight nucleotides starting at the 5′ end, is essential in target sequence recognition [2, 3]. Depending on the recognition site, binding the miRNA‐induced silencing complex to the cognate target can have different outcomes [4]. If the binding site is partially complementary, translation repression occurs, whereas when it is entirely complementary, the outcome is the degradation of the targeted transcript. miRNAs can be arranged in families with several members varying only by a few nucleotides outside the seed region. Genes of miRNA family members are generally clustered in chromosomal regions and present in one or more copies [7]. MicroRNAs (miRNAs) play a significant role in the development and progression of head and neck cancers, particularly OSCC. miRNAs can act as oncogenes (oncomiRs) or tumor suppressors, depending on their target genes [8]. Dysregulated miRNAs have been associated with various cancer hallmarks, such as proliferation, invasion, metastasis, and resistance to apoptosis [9, 10].

The article focuses on the biological roles and clinical relevance of miR‐181 family members in OSCC. It provides a comprehensive overview of current knowledge regarding their involvement in key tumor processes, including proliferation, invasion, metastasis, and therapy resistance, and evaluates their potential as diagnostic, prognostic, and predictive biomarkers. In addition, it examines the capacity of miR‐181 members to serve as therapeutic targets, highlighting how these transcripts interact with critical molecular pathways implicated in OSCC pathogenesis. By integrating data from experimental and clinical studies, the article also explores their potential utility in precision medicine strategies aimed at improving patient outcomes. The literature search was performed using the keywords “miR‐181a,” “miR‐181b,” “miR‐181c,” “miR‐181d,” and “OSCC.”

## 2 miR‐181 Family Members

The miR‐181 family members are highly conserved across species, suggesting potential functional redundancy and evolutionary importance. They are believed to have originated in urochordates, underscoring their deep evolutionary roots [11]. The conservation of miR‐181 across species underscores its essential role in regulating various biological processes, including transcriptional and translational regulation, signaling transduction [5, 11, 12, 13]. Members of the miR‐181 family typically have two paralogous gene clusters in most species. They share a conserved 5 “seed” sequence (nucleotides 2–8), which is critical for target recognition, while their 3 regions are more variable and contribute to target specificity and functional diversity [13, 14, 15]. There are six pre‐miRNAs of the miR‐181 family [11, 13]. The miR‐181 family consists of four mature miRNAs: miR‐181a, miR‐181b, miR‐181c, and miR‐181d. MiR‐181a/b are organized into two gene clusters: miR‐181a/b‐1 and miR‐181a/b‐2, located on different chromosomes in humans, 1 (1q32.1) and 9 (9q33.3), respectively. MiR‐181c/d is a cluster located on chromosome 19 (19p13.13). The expression patterns of miR‐181 family members exemplify context‐dependent gene regulation, as their levels are finely modulated across various tissues and pathological conditions. This dynamic regulation underscores their diverse functional roles, including the modulation of cellular differentiation, immune responses, and cancer progression. In OSCC, specific isoforms of miR‐181 have been shown to act either as tumor suppressors or oncogenes, depending on the molecular and cellular context.

**FIGURE 1:**
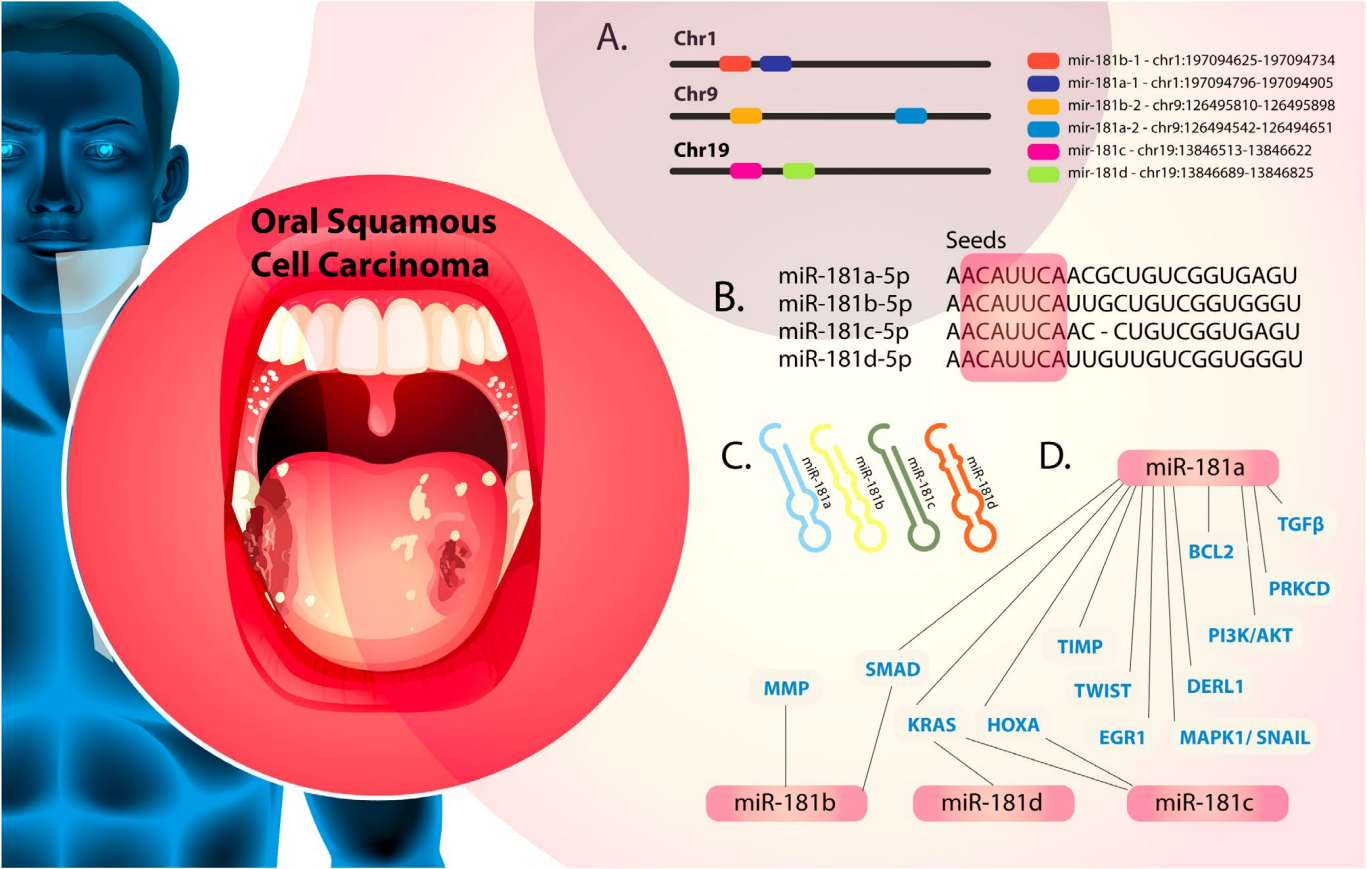
Summary of the role of the miR‐181 family members (miR‐181a, miR‐181b, miR‐181c, and miR‐181d) in OSCC. The blue color represents target genes influenced by these miRNAs, which are involved in critical processes such as apoptosis regulation, cell proliferation, metastasis, and chemoresistance. The interactions between miR‐181 family members and their target genes underscore their potential as diagnostic markers and therapeutic targets in OSCC.

Functional annotation of their target genes indicates that the miR‐181 family is involved in key biological processes and signal transduction pathways. The diverse regulatory network and context‐dependent roles of miR‐181 underscore the importance of investigating its expression patterns and upstream regulators, particularly transcription factors, to better understand its function in various malignancies. In this paper, we provide a comprehensive overview of the roles of the miR‐181 family in OSCC (Figure 1), focusing on their expression profiles and functional data from preclinical studies. This analysis aims to unravel the complex regulatory landscape governed by miR‐181 members in the OSCC tumorigenesis and disease progression.

## 3 miR‐181 Family Members' Expression Level in Tissue of Patients With Oral Cancers

Recent investigations have revealed that miR‐181 family members have altered expression levels in various tumor tissues and play a pivotal role in carcinogenesis and tumor progression (Table 1). The transcripts were shown to exhibit oncogenic or tumor‐suppressive properties in a cancer‐specific manner [26]. The dual‐role regulation of these miRNAs in the progression of human cancers generally depends on their mRNA targets and the main biological processes in which they are involved [27, 28, 29]. The role of these transcripts in cancer progression is largely determined by their specific mRNA targets and the biological processes they regulate [27, 28, 29]. As tumor suppressors, their capacity to reduce cell proliferation and the cell cycle has been documented in prostate cancer [30]. Their oncogenic role was shown in cervical cancer, where they were shown to promote cancer cell proliferation, invasion, and suppress apoptosis [31]. These examples demonstrate the complex and controversial roles of these transcripts in cancer biology.

**TABLE 1:** miR‐181 family members' expression levels in oral pathology.

Pathology	miRNA expression	Role	References
OL and OSCC	Increase of miR‐181a in OSCC (*n* = 17), OL (*n* = 22) when compared with normal oral mucosa tissue (*n* = 6)	Prognostic value, malignant transformation	[16]
OL and OSCC	Increase of miR‐181a in OSCC (*n* = 12) when compared to OL dysplasia (*n* = 22), normal oral mucosa (*n* = 6)	Prognostic value along with miR‐21, miR‐345	[17]
LSCC:	Increase of miR‐181a in LSCC tissue; actinic cheilitis without epithelial dysplasia (*n* = 19), actinic cheilitis with epithelial dysplasia (*n* = 32), and LSCC (*n* = 42) tissue	Early diagnostic marker involved in malignant transformation of squamous carcinoma	[18]
OSCC	Increase of miR‐181a/b in OSCC (*n* = 158) versus normal counterpart tissue (*n* = 158)	Part of a 4‐miRNA panel (miR‐21‐5p, miR‐107, miR‐1247‐3p, and miR‐181b‐3p)‐for lymph node metastasis;	[19]
OVC and OSCC	Increase of miR‐181b (30 well‐differentiated OSCC tumors and normal adjacent tissue; 15 normal oral mucosa samples)	Prognostic marker	[20]
OSCC	Decrease of miR‐181a in OSCC (*n* = 4) and normal adjacent tissue (*n* = 4)	Therapeutic target: suppressive effect by downregulating K‐ras.	[21]
OSCC	Decrease of miR‐181a in OSCC (*n* = 5) versus normal counterpart tissue (*n* = 3)	Therapeutic target and diagnostic biomarker	[1]
OSCC	Decrease of miR‐181a in OSCC (*n* = 15) versus normal counterpart tissue (*n* = 15)	Therapeutic target: promote tumor growth	[2]
OSCC	Decrease of miR‐181d in OSCC (*n* = 20) versus normal counterpart tissue (*n* = 20)	Therapeutic target	[22]
OVC and OSCC	Increase of miR‐181b (30 well‐differentiated OSCC tumors and normal adjacent tissue; 15 normal oral mucosa samples)	Prognostic marker	[20]
OSCC	Increase of miR‐181a OSCC (*n* = 39) versus normal counterpart tissue (*n* = 39); 39 blood samples from OSCC and 12 heathy controls	Correlated with lymph node metastasis, vascular invasion, and poor survival	[23]
OSCC	Increase of miR‐181b in controls (*n* = 10), low grade dysplasia (*n* = 10), high‐grade dysplasia (*n* = 10), OSCC (10)	Increased with increasing degree of dysplasia; discriminating between low‐grade and high‐grade dysplasia	[24]
OSCC	Decrease of miR‐181b, miR‐181c in saliva samples were collected from 6 cases with no dysplasia, 7 cases with low‐grade dysplasia, 10 cases with high‐grade dysplasia, and 10 cases with OSCC	Biomarker of dysplasia; miR‐181b/c was upregulated in high‐grade dysplasia, increasing with the degree of dysplasia and decreasing in OSCCs	[25]

Abbreviations: LSCC: lip squamous cell carcinoma; OSCC: oral squamous cell carcinoma; OSF: oral submucous fibrosis; OVC: oral verrucous carcinoma; SACC: salivary adenoid cystic carcinoma.

Some previous studies report that miR‐181 family members are downregulated in OSCC samples and cell lines [1, 21]. However, conflicting evidence exists, with some reports indicating overexpression of miR‐181a/b in OSCC compared to oral leukoplakia and normal oral mucosa [16, 17]. This conflicting evidence suggests that the role of miR‐181 family members in OSCC may be context‐dependent, influenced by factors such as disease stage or specific tumor microenvironment components [21, 22, 32, 33]. The potential of miR‐181 family members as molecular biomarkers in OSCC has sparked significant interest due to their differential expression in normal versus cancerous tissues and their relationship with disease progression and treatment response [34]. The potential of miR‐181 family members as molecular biomarkers in OSCC has sparked significant interest due to their differential expression in normal versus cancerous tissues [34]. Also, the role of miR‐181 family members in the tumorigenesis and progression of oral cancer was highlighted [17, 35]; a relationship was identified between the expression level of miR‐181 family and OSCC aggressiveness and poor prognosis.

miR‐181a and miR‐181d have been shown to have lower expression in OSCC tissues and cell lines than their normal counterparts [1]. This downregulation indicates their tumor‐suppressive role and utility in the early detection and prognosis of OSCC. miR‐181a and miR‐181d levels have been linked to tumor grade, stage, and metastatic potential, with lower levels indicating more aggressive disease phenotypes [19, 21, 35] and hold promising potential in detecting OSCC in non‐invasive diagnostic assays such as liquid biopsies.

A miRNA signature composed of miR‐21, miR‐18a, miR‐134a, miR‐210, miR‐181a, miR‐19a, and miR‐155 was associated with poor survival in HNSCC [36]. Overexpression of miR‐181b, miR‐31, and miR‐345 was observed in cheilitis without epithelial dysplasia, compared to actinic cheilitis with epithelial dysplasia and lower lip cancer. Therefore, these transcripts could be considered biomarkers of malignant transformation [18]. Another study revealed the overexpression of a miRNA signature composed of miR‐21, miR‐345, and miR‐181b in oral leukoplakia, a condition known to be associated with OSCC, emphasizing the role in malignant transformation [16]. Although OSCC may develop from oral leukoplakia, clinical and histological evaluation markers have limited predictive value in predicting which leukoplakic lesions carry a higher risk of malignant progression [17]. miR‐21 and miR‐181b expression patterns were associated with certain cytological and histopathological features, such as increased mitotic figures, nuclear/cytoplasmic ratio, or hyperchromasia [17].

Oral submucous fibrosis is a chronic fibrotic remodeling disease that can lead to oral cancer. A recent study emphasized the important role of the MEG3/miR‐181a axis in this process. This finding underscores the crucial role of the MEG3/miR‐181a axis in disease progression and its potential as a therapeutic target in preventing the transformation of fibrotic lesions into malignant tumors [37].

Oral verrucous carcinoma is characterized by a low proliferation rate and a high degree of differentiation, with a high prevalence in male smokers [20]. miR‐181b was overexpressed in oral verrucous carcinoma and OSCC compared to normal tissues; the expression levels for this transcript in oral verrucous carcinoma samples significantly decreased compared to OSCC samples [20]. The expression level of miR‐181b in peritumoral samples was relatively lower compared to tumor tissue, highlighting its important role in tumorigenesis and malignant progression [20]. Moreover, high‐risk human papillomaviruses (e.g., HPV16 and HPV18) are associated with the development of OSCC. HPV16‐mediated E6 degradation of p53 inhibits the expression of miR‐181a and miR‐181d, supporting the functional importance of the HPV16/miR‐181a/d axis in HPV‐mediated oral carcinogenesis [38].

Senescence is a key mechanism for tumor suppression. Increased levels of miR‐181a were observed during replicative senescence of normal human oral keratinocytes. Furthermore, the effect of miR‐181a overexpression on senescence was evaluated in OSCC. The ectopic expression of miR‐181a was related to the inhibition of cell proliferation and anchorage‐independent growth ability of OSCC cells [21].

A recent study found that the knockdown of MEG3 was related to reducing myofibroblast activities, which were reestablished by inhibiting miR‐181a and overexpressing the *EGR1* gene [37]. miR‐181a‐5p was downregulated in salivary adenoid cystic carcinoma and correlated with upregulated circ‐001982. circFNDC3B sequestered miR‐181c‐5p to upregulate SERPINE1 and PROX1, which drove epithelial–mesenchymal transition (EMT) mechanisms and promoted lymphangiogenesis to accelerate lymph node metastasis in OSCC [39].

Metastasis to lymph nodes in the neck is a crucial sign of tumor spread in OSCC patients and an important prognostic factor. For patients with occult metastasis, there is an urgent need to develop novel biomarkers to improve disease management [19]. In this regard, a 4‐miRNA panel (miR‐21‐5p, miR‐107, miR‐1247‐3p, and miR‐181b‐3p) was identified to discriminate the nodal disease, revealing a predictive value for lymph node metastasis in OSCC [19]. Among various clinical variables, overexpression of miR‐181 was significantly correlated with lymph node metastasis, vascular invasion, and poor overall survival [23]. Functional assays demonstrated that ectopic overexpression of miR‐181 enhanced cell migration and invasion in OSCC cells, without affecting their anchorage‐independent growth. Notably, overexpression of miR‐181 was detected both in tumor tissues and in plasma samples, suggesting its potential as a circulating biomarker [23].

## 4 Expression of miR‐181 Family Members in the Plasma and Saliva of Patients With Oral Cancers

In the context of OSCC, non‐invasive and easily accessible biofluids such as plasma and saliva represent valuable sources for miRNA‐based biomarker discovery. Unlike tissue biopsies, which are invasive and provide only a snapshot of tumor heterogeneity, liquid biopsies offer a dynamic and systemic view of disease progression and response to treatment.

Yang et al. demonstrated that miR‐181 is significantly up‐regulated in both tumor tissues and plasma of OSCC patients, with elevated levels correlating with lymph‐node metastasis, vascular invasion, and poor survival. Functional assays revealed that ectopic overexpression of miR‐181 enhances OSCC cell migration and invasion, though not anchorage‐independent growth. These findings suggest miR‐181 as a potential biomarker and contributor to OSCC metastasis [23]. Also, miR‐181 was identified as part of a group of 18 microRNAs showing distinct expression patterns between metastatic and non‐metastatic OSCC. While miR‐31 and miR‐130b were associated with non‐metastatic cases, miR‐181 and miR‐296 were linked to metastasis. Notably, miR‐181 was detected exclusively in metastatic tumors and in the plasma of patients with metastasis, underscoring its potential as a non‐invasive biomarker. Its consistent presence in both tissue and plasma highlights its value for identifying and monitoring metastatic disease [40].

Saliva is of high interest in oral cancer research due to its direct contact with oral epithelial lesions and its content of extracellular vesicles and cell‐free nucleic acids derived from both local and systemic sources. Similarly, circulating miRNAs in plasma are reflective of tumor‐secreted molecules and systemic alterations induced by malignancy, making them promising candidates for early detection and monitoring.

The expression of miR‐181b has been examined in the context of oral dysplasia and carcinoma progression, often revealing dynamic and stage‐dependent changes. It was demonstrated that salivary levels of miR‐27b and miR‐181b progressively increased with the severity of epithelial dysplasia, reaching the highest levels in high‐grade dysplasia cases. Interestingly, a marked reduction in miR‐181b levels was observed in samples from OSCC patients, suggesting that while miR‐181b may act as an oncogenic signal during early dysplastic transformation, it could be suppressed or downregulated as malignancy fully develops, possibly reflecting shifts in tumor microenvironment or cellular differentiation states [24]. In contrast, another recent investigation analyzing the salivary miRNA profiles of patients with leukoplakia found that miR‐181b and miR‐181c were downregulated in cases of low‐grade dysplasia (LGD) that eventually progressed to more severe forms [25]. This finding contradicts earlier reports of miR‐181 upregulation and underscores the complexity and context‐dependency of miRNA behavior during oral carcinogenesis. Such discrepancies may arise due to variations in patient populations, methods of sample processing, or temporal stages of lesion progression, highlighting the need for standardized longitudinal studies. Altogether, these observations support the idea that miR‐181 isoforms exhibit a biphasic expression pattern during oral cancer development, with potential implications for early detection, risk stratification, and monitoring disease progression. Further studies involving both plasma and saliva specimens in larger, well‐defined cohorts are warranted to clarify the diagnostic value and biological significance of miR‐181 family members in OSCC.

## 5 miR‐181 Family Members in Preclinical OSCC Studies

The miR‐181 family has been extensively studied in various preclinical models of OSCC, revealing its complex involvement in the disease's pathology. Each miR‐181 member was demonstrated to target different genes, leading to varied functionality and context‐sensitive activities. Table 2 summarizes the complex biological properties of these transcripts in OSCC cell lines and animal model studies. Several studies have demonstrated that miR‐181 family members act as tumor suppressors during OSCC tumorigenesis and development, which supports the downregulated levels in OSCC saliva [1, 24]. Restoring the expression levels of miR‐181a‐5p was related to reducing cell proliferation, colony formation, invasion, and migration. This also blocked the cell cycle and promoted apoptosis [1]. Moreover, although specific miR‐181a expression levels were not reported in oral submucous fibrosis, primary normal and fibrotic buccal submucous fibroblasts showed fucoidan‐mediated inhibition of fibrotic properties via the MEG3/miR‐181a/Egr1 axis, highlighting the therapeutic potential of targeting this pathway [37]. In adenoid cystic carcinoma, the metastatic SACC‐LM cell line showed significantly lower miR‐181a expression than the less aggressive SACC‐83 cell line [41]. This decrease coincided with an increase in circRNA_001982, indicating that miR‐181a plays a role in metastatic processes.

**TABLE 2:** Preclinical studies evaluating the role of miR‐181 family members in oral pathology.

Pathology	Decreased expression levels	Experimental model	Delivery system	Observation	References
OSF	N/A	Primary normal and fibrotic buccal submucous fibroblasts	Lipofectamine 2000	Fucoidan‐mediated inhibition of fibrotic properties in OSF via the MEG3/miR‐181a/Egr1 Axis	[37]
Adenoid cystic carcinoma	miR‐181a (SACC‐LM vs. SACC‐83)	SACC‐83 and SACC‐LM	Lipofectamine 2000	Correlation with circRNA_001982 upregulation, involved in metastasis	[41]
OSCC	miR‐181a	SCC‐9, KB, Cal‐27, SCC‐25, HN6 and SCC‐090	Lipofectamine 3000	miR‐181a‐mediated Wnt/β‐catenin signaling, mediating invasion and metastasis	[2]
OSCC	miR‐181a	SCC7, SCC25, Cal27 and C2C12	Lipofectamine3000	miR‐181a‐3p‐regulated TERS signaling from OSCC cell lines to induce C2C12 myotube atrophy and apoptosis	[42]
OSCC	—	CAL27 and SCC15	Lipofectamine 2000	miR‐181a directly targeted Twist1 and then reversed chemoresistance and EMT	[34]
OSCC	—	CAL‐27, SCC‐9, CHO, SH‐SY5Y and HT22	miR‐181a‐5p/AgNPs complexes	Decrease cell growth and progression in vitro and in vivo	[43]
OSCC	miR‐181d	SCC‐4, SCC‐9, CAL‐27 and Ca9‐22	Lipofectamin RNAiMAX	Affect cell viability and apoptosis in OSCC cells	[22]
OSCC	miR‐181a	Organotypic raft culture	miR‐181a precursor, Letiviral delivery, LV‐GFP	miR‐181a was upregulated during replicative senescence of normal human oral keratinocytes miR‐181a shows tumor suppressive effect against oral squamous cell carcinoma cells by downregulating KRAS	[21]
OSCC	miR‐181a	CAL‐27 and SCC‐25, nude mice	Lipofectamine, RNAiMAX	Inhibits cell invasion and migration	[1]
OSCC/oropharyngeal SCC	miR‐181a/d in HPV‐infected cells.	SCC66, SCC105, UM6, UM10b, in vivo xenograft tumor assay	Pre‐miR‐181a and 181d (Ambion), Lipofectamine 2000, Scramble oligonucleotides	HPV16 inhibited the expression of miR‐181a and miR‐181d (miR‐181a/d) miR‐181a/d may represent a novel therapeutic agent for the treatment of HPV‐positive OSCC. Possible involvement of p53 in the regulation of miR‐181a/d because HPV16 suppresses the expression of miR‐181a/d in oral keratinocytes containing wild‐type p53 (data not shown) and the OSCC cell lines containing the defective p53 gene	[38]
SACC	miR‐181a	SACC83/SACC‐LM and xenograft nude mice	Lentiviral delivery	Regulate MAPK/SNAI2 pathway related to invasion and proliferation migration and metastatic processes	[44]
OSCC	—	HSC3 and CAL27, HUVEC, HLEC, and xenograft nude mice	Lipofectamine 2000	circFNDC3B sequestered miR‐181c‐5p to upregulate SERPINE1 and PROX1, regulating EMT, invasion and metastasis	[39]

In studies focusing on HPV‐infected oral/oropharyngeal squamous cell carcinoma, miR‐181a expression was significantly reduced in the cell lines SCC66, SCC105, UM6, and UM10b [38]. In vivo, xenograft assays with pre‐miR‐181a and 181d delivered via Lipofectamine 2000 revealed that HPV16 suppresses the expression of these miRNAs, possibly via a p53‐dependent mechanism. This suppression suggests that miR‐181a/d could be used as new therapeutic agents for HPV‐positive OSCC, particularly given their role in downregulating KRAS and exhibiting tumor‐suppressive properties. Furthermore, miR‐181a precursor delivered via lentiviral vectors in organotypic raft cultures showed that miR‐181a is upregulated during replicative senescence of normal human oral keratinocytes, confirming its tumor‐suppressive properties [21]. Additional studies on OSCC cell lines revealed that low miR‐181d expression reduces cell viability and promotes apoptosis [21, 22]. Furthermore, miR‐181a downregulation in CAL‐27 and SCC‐25 cells and in nude mouse models significantly reduced cell invasion and migration [1]. Notably, miR‐181a‐3p was found to regulate TERS signaling from OSCC cell lines, resulting in C2C12 myotube atrophy and apoptosis [42]. Another study found that miR‐181a regulates the WNT/β‐catenin signaling pathway, which promotes cell invasion and metastasis [2]. Similarly, in salivary adenoid cystic carcinoma models, lower miR‐181a expression in SACC83 and SACC‐LM cells and xenograft nude mice influenced the MAPK/SNAI2 pathway, affecting invasion and proliferation [44]. Interestingly, circFNDC3B was found to sequester miR‐181c‐5p, which upregulates SERPINE1 and PROX1, promoting EMT, invasion, and metastasis [39].

Various preclinical studies have evidenced that miR‐181 family members, particularly miR‐181a and miR‐181d, play important regulatory roles in OSCC pathogenesis and influence the cellular processes. The overexpression of miR‐181d, an oncogenic driver, has been shown to reverse the inhibitory effects of miR‐181d mimics on OSCC cell proliferation and growth [21, 22]. Future research should focus on elucidating the regulatory mechanisms governing the expression of miR‐181 family members and their interactions with key oncogenic pathways, which will pave the way for their potential establishment not only as therapeutic targets but also as biomarkers.

## 6 Chemoresistance and Radioresistance

Chemoresistance in OSCC represents significant therapeutic obstacles, contributing to poor clinical outcomes and high recurrence rates. These phenomena are primarily driven by a range of genetic, epigenetic, and molecular alterations that enable tumor cells to survive cytotoxic insults and evade therapeutic interventions. Despite ongoing research efforts, the underlying mechanisms responsible for chemoresistance in OSCC remain incompletely understood, highlighting the complexity of this multifaceted process. Additionally, radioresistance often shares overlapping mechanisms with chemoresistance, including DNA repair enhancement, apoptosis inhibition, and activation of pro‐survival signaling pathways.

In general, platinum‐based drugs are still the most widely used agents in chemotherapy for OSCC. However, research on miR‐181a‐5p's role in platinum drug resistance in OSCC is limited; growing evidence from other epithelial tumors suggests its involvement in chemoresistance. In cisplatin‐resistant lung squamous cell carcinoma (SCC) cells, reduced levels of miR‐181a‐5p have been linked to the increased expression of the oncogene CUGBP Elav‐like family member (CELF1) [45]. Additionally, DLX6‐AS1 was found to interact with both miR‐181a‐5p and miR‐382‐5p to regulate CELF1 expression, thereby influencing cisplatin sensitivity in resistant lung SCC cells. This indicates a complex regulatory mechanism involving miR‐181a‐5p in chemoresistance across different cancers [45]. Other targets of miR‐181a‐5p by which it increases chemosensitivity to platinum drugs have also been found, including the vitamin D receptor in breast cancer cells [46], CBLB in esophageal adenocarcinoma [47], and GRP78 in cervical cancer [48]. The effect of melatonin on cisplatin‐induced cell death and Derlin‐1 (DERL1) endoplasmic reticulum membrane protein expression has recently been evaluated. It was revealed that miR‐181c‐5p expression was significantly upregulated in the presence of melatonin; melatonin‐induced miR‐181c‐5p enhances cisplatin‐induced cell death through inhibition of DERL1 in mesenchymal‐like CD44^high^ cells [49]. A study on both cervical cancer lines and mouse tumor xenograft models found that higher miR‐181a levels were associated with resistance to radiotherapy, an effect mediated by the downregulation of the pro‐apoptotic *PRKCD* gene by miR‐181a [50]. In tongue squamous cell carcinoma (TSCC), chemoresistance has been linked to EMT activation and increased metastatic potential mediated through the miR‐181a/TWIST1 signaling axis [34].

Cetyltrimethylammonium bromide has been shown to affect the mesenchymal characteristics of SCC4 cells by regulating the TGF‐β/SMAD/miR‐181b/TIMP3 axis, leading to extracellular matrix remodeling [51]. This suggests it could be a promising antimetastatic therapeutic agent for TSCC, via TFs. This pathway is tightly regulated by transcription factors (TFs), particularly SMADs, which act downstream of TGF‐β signaling to control miR‐181b expression. The resulting downregulation of TIMP3, an inhibitor of matrix metalloproteinases, facilitates ECM degradation and promotes metastatic potential. These findings suggest that CTAB, through its influence on TF‐mediated signaling, could serve as a promising antimetastatic therapeutic agent by targeting the transcriptional control of ECM remodeling [51].

However, further studies are needed to better understand their mechanisms and potential therapeutic applications in overcoming drug resistance—invasion, migration, and metastasis. Invasion, migration, and metastasis are critical processes in cancer progression. Invasion refers to the ability of cancer cells to penetrate surrounding tissues, migration involves the movement of these cells to distant sites, and metastasis is the formation of secondary tumors at new locations. These processes are often driven by changes in the tumor microenvironment, including alterations in cell adhesion, matrix remodeling, and activation of signaling pathways like EMT. Targeting these pathways is crucial for preventing the spread of cancer and improving patient outcomes. miR‐181 family members have been implicated in regulating these processes by targeting key pathways such as the EMT and extracellular matrix remodeling [12, 52]. A recent study revealed that *CCAT1* lncRNA activated Wnt/β‐catenin signaling by decreasing the expression levels of miR‐181a, promoting the activation of cell proliferation, migration, and invasion of OSCC [2]. Another study revealed that miR‐181a deregulation mediated the metastasis of SACC by regulating the MAPK/SNAI2 pathway [44]. This signaling pathway plays a crucial role in cancer cell invasion by regulating EMT.

Inhibition of circRNA‐001982 inhibited migration and invasion. In contrast, miR‐181a‐5p up‐regulation had the opposite effect, as shown by wound healing and transwell assays. circRNA‐001982/miR‐181a‐5p is related to invasion and metastasis mechanisms [41]. Additionally, ectopic expression of miR‐181a/d in HPV16‐transfected OSCC reduced anchorage‐independent growth and the cancer stem cell (CSC) phenotype. This effect was achieved by inhibiting miR‐181a/d target genes, including *KRAS* and *ALDH1*, key elements in OSCC carcinogenesis. By suppressing these targets, the oncogenic effects of HPV16 in HPV16‐transfected OSCC were effectively neutralized [38]. This suggests that miR‐181a/d is critical in counteracting HPV16‐driven tumorigenesis in OSCC.

Most studies focused on miR‐181a, highlighting its involvement in key oncogenic processes such as apoptosis, cell survival, proliferation, and metastasis. This member has been linked to various molecular targets, including BCL2, PRKCD, and PI3K/AKT, making it a central player in chemoresistance, tumor growth, and metastasis. Due to its prominence in these critical cancer pathways, miR‐181a has emerged as a potential biomarker for diagnosis and a therapeutic target. However, it is essential not to underestimate the importance of other miR‐181 family members. Despite sharing a common seed sequence, these transcripts can have different biological effects due to their interaction with diverse genes. For instance, miR‐181b regulates extracellular matrix degradation and metastasis through MMP and inhibits metastasis via SMAD and TGFβ signaling. These distinct functions suggest that miR‐181b may offer therapeutic potential in blocking metastasis. Similarly, miR‐181c is involved in cell differentiation through targets like HOXA, providing opportunities to explore its role in therapeutic strategies to regulate cancer cell differentiation and self‐renewal. The limited number of studies on miR‐181b, miR‐181c, and miR‐181d compared to miR‐181a should not lead to their marginalization. Emerging evidence suggests that these members could play crucial yet distinct roles in regulating tumor behavior, offering new avenues for therapeutic intervention. As research advances, a more comprehensive understanding of each miR‐181 family member's unique contribution to cancer biology is critical (Table 3). This will help harness the full potential of these miRNAs in developing precision medicine strategies, not only in OSCC but also to be extended to other cancer types.

**TABLE 3:** The main implications of miR‐181 family members in diagnosis and therapy.

miR‐181 family member	Target genes in OSCC	Role in OSCC	Implication in diagnosis/therapy	References
miR‐181a	BCL2	Regulator of apoptosis, promotes cell survival	Diagnostic marker for survival‐related gene therapies	[1, 20, 43]
miR‐181a	PRKCD	Enhances chemoresistance through apoptosis reversion	Candidate for therapies targeting apoptosis and drug resistance	[50]
miR‐181a	DERL1	Promote cancer cell survival and immune evasion	Potential therapeutic target for inhibiting cancer cell survival	[49]
miR‐181a miR‐181c	KRAS	Oncogenic pathway drives cell survival and proliferation	Therapeutic target for blocking oncogenic signaling	[21, 22, 33]
miR‐181a	PI3K/AKT	Oncogenic pathway drives cell survival and proliferation	Therapeutic target for blocking oncogenic signaling	[53]
miR‐181a	TWIST	EMT regulator, invasion and metastasis; drug resistance	Diagnostic and prognostic markers for regulating EMT in therapies	[34, 54]
miR‐181b	TGFβ, TIMP3	Inhibitor of metalloproteinases regulates extracellular matrix	Candidate for antimetastasis therapy	[51]
miR‐181a	EGR1	Cell proliferation, invasion, and metastasis; drug resistance	Prognostic value; potential therapeutic target for inhibiting cancer cell survival	[55]
miR‐181a	MAPK1/SNAIL	Oncogenic pathway promotes cell survival and proliferation	Diagnostic marker for promoting survival and therapy resistance	[44]
miR‐181a miR‐181c	HOXA	Involved in cell differentiation	Potential target for cell differentiation modulation in therapies	[56, 57]

## 7 Conclusions

**FIGURE 2:**
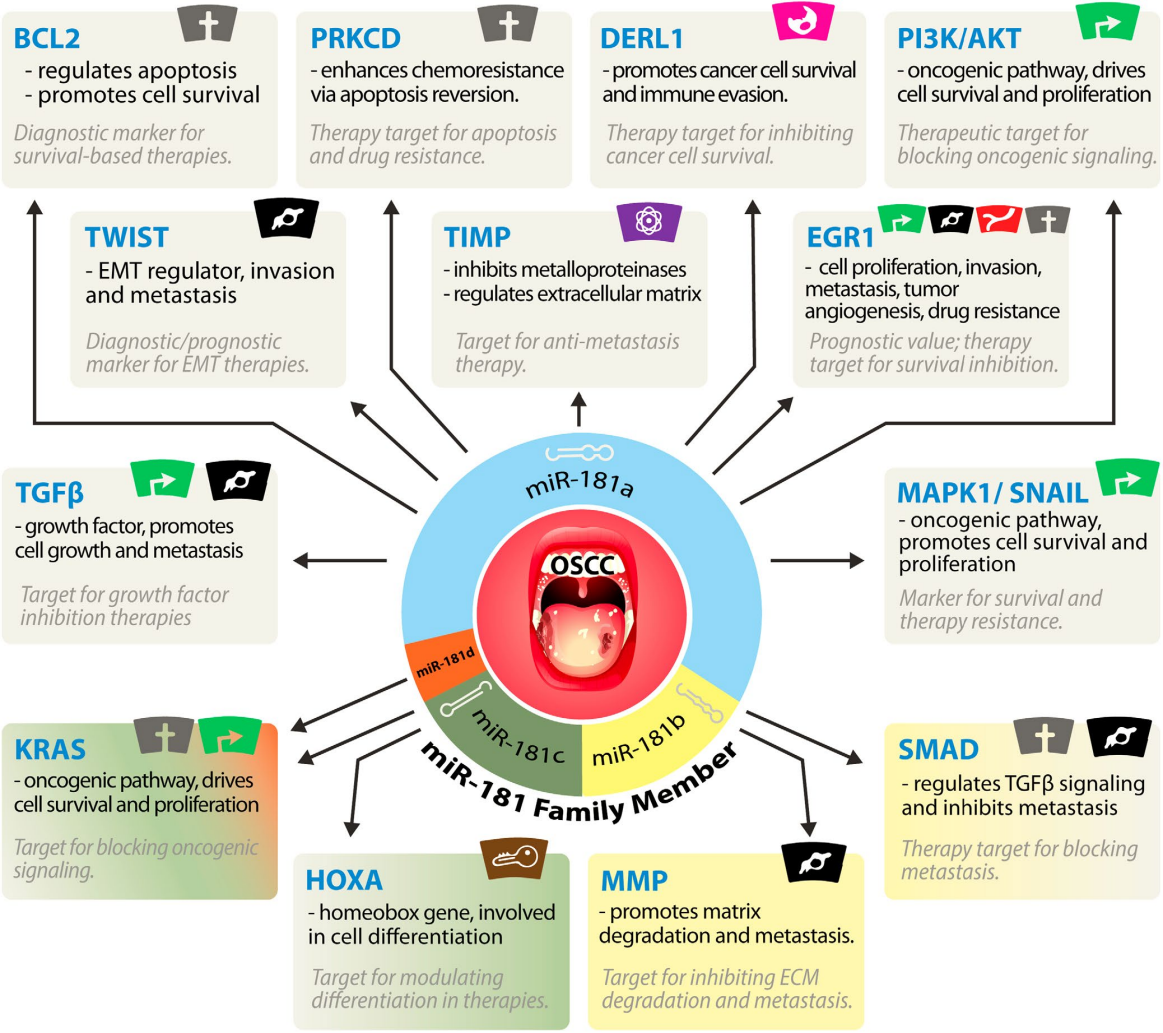
The Role of miR‐181 Family Members in Regulating Key Oncogenic Pathways in OSCC. This figure illustrates the involvement of miR‐181 family members (miR‐181a, miR‐181b, miR‐181c, and miR‐181d) in the pathogenesis of OSCC by regulating multiple target genes (depicted in blue). These genes are implicated in key cancer‐related processes such as apoptosis regulation, cell proliferation, metastasis, and chemoresistance, each contributing to activating specific hallmarks of cancer. The interactions between miR‐181 family members and their target genes suggest the miRNAs' dual role as diagnostic markers and therapeutic targets. Genes involved in survival‐based therapies, drug resistance, metastasis inhibition, and oncogenic signaling are highlighted, with their respective therapeutic implications indicated in gray italic text. The figure underscores the potential of miR‐181 as a central player in modulating cancer hallmarks, including resistance to cell death, sustained proliferation, invasion, and metastasis.

miR‐181a plays a pivotal role in regulating key genes involved in cell survival and oncogenic signaling, acting as a crucial modulator in the progression of OSCC. The data presented underscore that the miR‐181 family members, particularly miR‐181a, target and alter the expression levels of critical genes and pathways essential for promoting and maintaining the neoplastic state, including those linked to apoptosis, proliferation, metastasis, and resistance to therapy. Given the differential expression of miR‐181a in preneoplastic and neoplastic diseases, additional studies focusing on the regulatory role of miR‐181a‐5p are crucial (Figure 2). Understanding how miR‐181a is overexpressed in preneoplastic lesions yet downregulated in fully developed OSCC could provide key insights into early detection strategies and the transition from benign to malignant states. Moreover, studies should explore the potential use of miR‐181a modulation as part of a combinatorial therapy to enhance the efficacy of conventional OSCC treatments, such as chemotherapy and radiotherapy. Future investigations should also focus on validating the clinical utility of miR‐181 family members in non‐invasive diagnostic methods, such as analyzing miR‐181 expression levels in biofluids like plasma and saliva. This could lead to the development of miRNA‐based diagnostic assays for early detection and monitoring of OSCC progression and response to therapy.

In conclusion, while significant progress has been made in understanding the role of miR‐181a in OSCC, comprehensive research into the entire miR‐181 family is warranted. Unraveling the complex regulatory networks involving these miRNAs could lead to novel therapeutic strategies targeting the molecular underpinnings of OSCC, ultimately improving patients' outcomes.

## Author Contributions

**Alexandra Iulia Aghiorghiesei:** conceptualization, investigation, writing – original draft, validation, methodology, data curation. **Andreea Nutu:** investigation, validation, methodology, visualization, software, formal analysis, data curation, writing – original draft. **Nikolay Mehterov:** investigation, validation, writing – review and editing, supervision, data curation, formal analysis. **Christos K. Kontos:** investigation, validation, visualization, writing – review and editing. **Boyan Vladimirov:** visualization, formal analysis, data curation, investigation. **Rares Buduru:** investigation, validation, visualization, methodology, data curation, writing – review and editing. **Cornelia Braicu:** conceptualization, investigation, writing – original draft, validation, visualization, formal analysis, supervision, data curation, writing – review and editing. **Ioana Berindan‐Neagoe:** conceptualization, writing – review and editing, visualization, methodology, project administration, formal analysis, data curation, supervision.

## Conflicts of Interest

The authors declare no conflicts of interest.

## Data Availability

Data sharing not applicable to this article as no datasets were generated or analysed during the current study.
